# Glycitin in Soy Beans: A Mine of Their Structures, Functions, and Potential Applications

**DOI:** 10.3390/foods14172940

**Published:** 2025-08-23

**Authors:** Hongqiang Wu, Yanyu Feng, Xianyang Xie, Yi Zhang

**Affiliations:** College of Food Science, Fujian Agriculture and Forestry University, Fuzhou 350002, China; wuhongqiang1010@163.com (H.W.); fengyanyu1221@163.com (Y.F.); xjyyy317@163.com (X.X.)

**Keywords:** isoflavones, soybean, glycitin, glycitein, biological activities

## Abstract

Glycitin is a kind of compound found in soybeans that has attracted increasing attention as a good source of nutrients due to its potential applications in medicine, cosmetics, and food. However, a comprehensive review of glycitin is lacking to help readers understand the current state of research on isoflavones. The present review summarizes recent progress made on the structures and functions of glycitin, with future perspectives to maximize their value and applications using bibliometric analysis methods. The health effects attributed to glycitin include estrogenic effects and anti-osteoporosis effects, as well as antioxidant, anti-tumor, hypolipidemic, and hypoglycemic effects. This review can not only deepen the understanding of the functions of glycitin, but also lay an important foundation for the further development and utilization of soybean resources.

## 1. Introduction

Soybean is important and the world’s fourth-largest crop. They serve as a rich source of various bioactive substances, including proteins, in the human diet. Soy isoflavones (SI) are physiologically active flavonoid compounds produced by soybeans during their growth process. They can be classified into three major groups: daidzin groups, genistin groups, and glycitin groups [1]. Research has shown that soy isoflavones exhibited multiple physiological activities, including antioxidant effects [2], bidirectional regulation of estrogen [3,4], anti-osteoporosis properties [5], anti-tumor effects [6], anti-aging effects [7], neuroprotective effects [6], regulation of cell proliferation and apoptosis [8], and modulation of glucose and lipid metabolism [9]. Although there have been numerous studies on the composition and biological activities of soy isoflavones, a review specifically focusing on the functional activities of glycitin is relatively limited. Therefore, a basic understanding of the structural characteristics and biological activities is crucial for the successful application of glycitin and maximizing its value. As discussed in this review, the application of glycitin in food also seems to face technical challenges and limitations. Bibliometric science mapping is a quantitative method that analyzes scientific publications throughout the terms present in their title, abstract, and keywords, which is a powerful tool to observe trends and identify research opportunities. Bibliometrics scientific mapping has been applied in many fields, and also in food. Therefore, this review provides an overview of the isolation, structural, and functional properties of glycitin with the aid of bibliometric analysis methods for the first time [10]. The type of presentation contained the bar chart of the number of publications on glycitin, the bar chart of publication types on glycitin, the tree map chart of journals on glycitin, the term clustering map of publications on glycitin, and the tree map chart of research fields on glycitin.

## 2. Bibliometric Analysis

**Figure 1:**
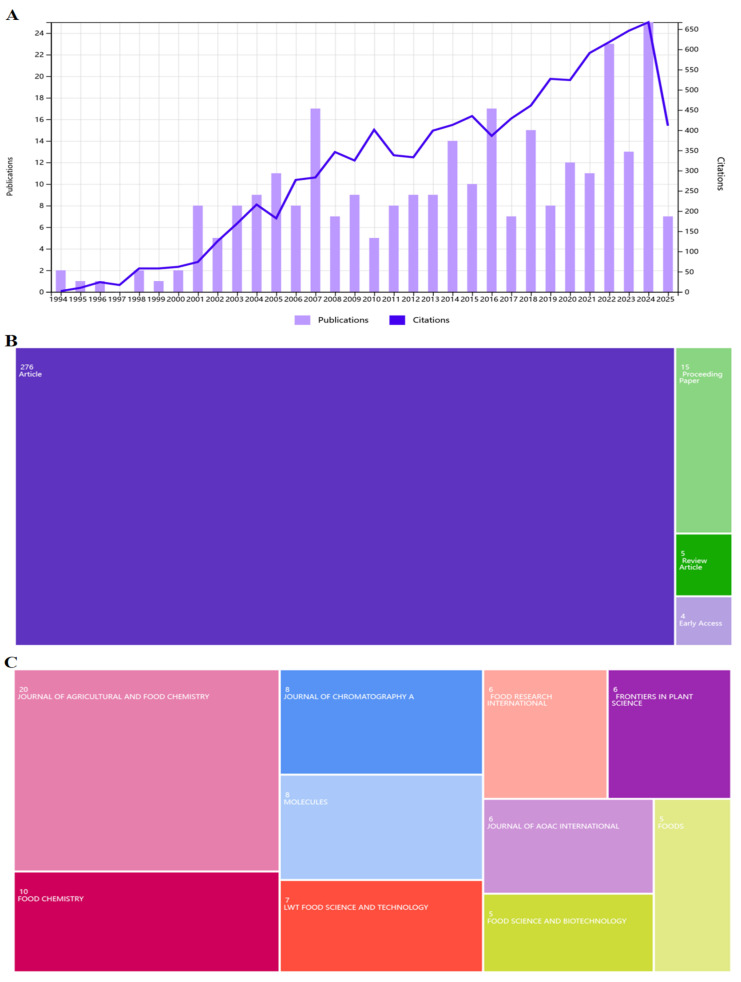

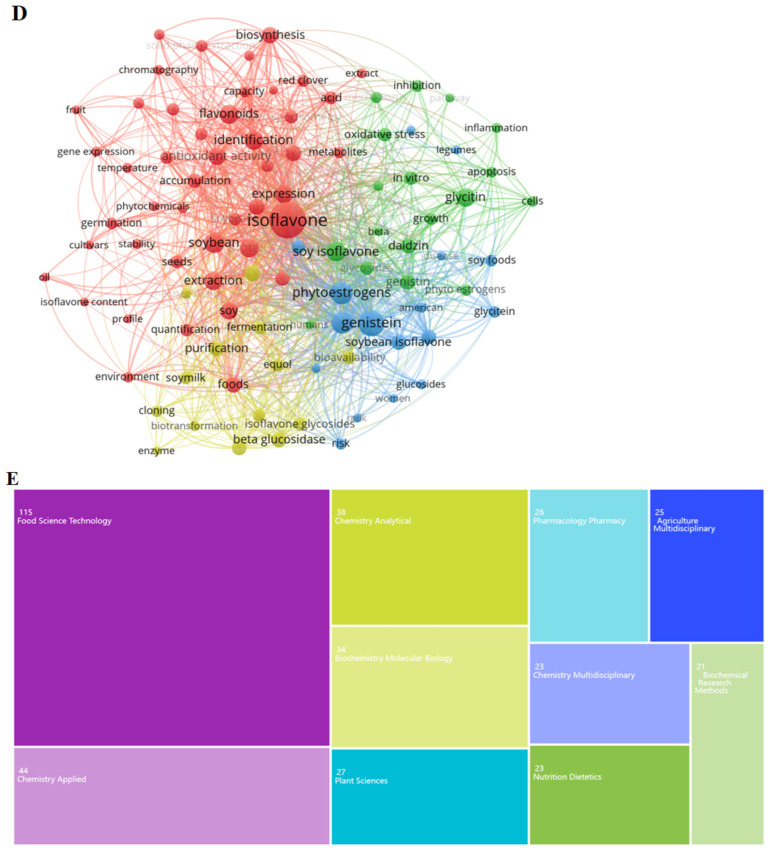
The number, type, journals, research terms, and fields of publications on glycitin based on the Web of Science. (**A**) The bar chart of the number of publications on glycitin. (**B**) The tree map chart of publication types on glycitin. (**C**) The tree map chart of journals on glycitin. (**D**) The term clustering map of publications on glycitin. Different colors represent the terms that belong to different clusters. The size of the term is based on the number of occurrences. (**E**) The tree map chart of research fields on glycitin.

“Glycitin” was selected as the keyword to retrieve related research during the period of 1974–2025 on the Web of Science database. The searches were restricted to English publications (articles, review articles, abstracts, and meetings, etc.). Bibliometrix was used for bibliometric analysis, and a term clustering map was created by using VOSviewer software 1.6.20 (Leiden University’s Centre for Science and Technology Studies, Leiden, the Netherlands). Figure drawn using ChemDraw 23.1.1.3 (PerkinElmer, Waltham, MA, USA). A total of 284 scientific publications related to glycitin were selected from the database, with the highest number of publications obtained in 2024. The number of citations increased each year and reached its peak in 2024 (Figure 1A). Among the selected publications, a total of 276 scientific publications played an absolute dominant role as the core carrier of knowledge output, showing the main achievements of basic research and application exploration of glycitin. A total of 15 scientific publications provided a platform for the exchange of academic conference outcomes, facilitating the immediate sharing of research trends. A total of five review articles were crucial for sorting out research threads and integrating knowledge systems. A total of four early-access articles represented the rapid release of cutting-edge achievements, reflecting the timeliness of field research (Figure 1B). The most published journals were the *Journal of Agricultural and Food Chemistry* (7.04%) and *Food Chemistry* (3.52%), followed by the *Journal of Chromatography A* (2.82%), among others (Figure 1C). These published journals have become important windows for researching glycitin in the fields of food chemical characteristic analysis and chromatographic detection technology applications. The word term clustering map (Figure 1D) displayed 95 terms grouped into 4 clusters. The red cluster, “isoflavones”, as the core anchor point, was associated with 22 terms such as “soybean”, “flavonoids”, and “antioxidant activity”, focusing on the plant sources and basic characteristics of isoflavones. The green cluster contained 17 terms, focusing on topics such as “apoptosis”, “inhibition”, “cancer”, and exploring the medical functions of isoflavones. The blue cluster (16 terms) gathered “genistein” and “phytoestrogens” to explore component identification and activity mechanisms. The yellow cluster (13 terms) contained topics such as “soy”, “foods”, “extraction”, etc., which were related to food applications and processes. There were also groups around terms such as “glycitin”, with different clusters interwoven, presenting a multidimensional research landscape of glycitein from plant metabolism and component identification to functional applications and medical potential, reflecting its rich interdisciplinary research connections. Among 284 scientific publications, its research scope contained multiple disciplinary fields (Figure 1E). The field of food science and technology reached the highest number of publications (115 scientific publications, 40.5% of the total publications). A total of 82 scientific publications in applied chemistry (44 scientific publications, 15.5% of the total publications) and analytical chemistry (38 scientific publications, 13.4% of the total publications) played a key role in the research and development of extraction, detection, and other technologies for glycitin. The fields of biochemistry and molecular biology (34 scientific publications, 12% of the total publications), plant science (27 scientific publications, 9.5% of the total publications), and pharmacology and pharmacy (26 scientific publications, 9.2% of the total publications) also played an important positions, promoting in-depth research in molecular mechanisms, plant sources, medicinal potential, and other directions. In addition, significant achievements have been made in fields such as multidisciplinary agriculture (25 scientific publications, 8.8% of the total publications), multidisciplinary chemistry (23 scientific publications, 8.1% of the total publications), nutrition (23 scientific publications, 8.1% of the total publications), and biochemical research methods (21 scientific publications, 7.4% of the total publications). These scientific publications indicated that glycitin has gained attention from the academic community due to its enormous potential application value in food development, drug research and development, and other fields.

## 3. Extraction and Purification of Glycitin

Naturally occurring biologically active substances are typically found in small quantities, and other compounds are easily extracted during the extraction process, resulting in difficulties in isolation and purification. Thus, all the pre-treatment methods precede the extraction methods, including microwave-assisted extraction, ultrasonic-assisted extraction, and pressurized solution extraction, with the aim of removing interfering compounds as much as possible before targeted extraction in the laboratory [11]. The extraction method of glycitin is similar to other isoflavone extraction methods, which usually use methanol, ethanol extract with acetonitrile, or their mixture of water. At present, the extraction methods of glycitin include hot water extraction and organic solvent extraction. However, a key challenge in obtaining glycitin from soy is its low extraction rate, and the extraction rate of glycitin is mostly less than 60% in the laboratory [12]. In the extraction process of glycitin, the existence of water-soluble substances, such as proteins and carbohydrates, is found to greatly affect the extraction rate. To overcome these challenges, suitable pretreatment methods or new extraction techniques should be employed to improve the recovery of glycitin. Current methods used for the isolation of glycitin are summarized in Table 1 from the laboratory. Although microwave-assisted extraction, ultrasound-assisted extraction, and pressurized solution extraction have better extraction effects and higher extraction efficiency, they are costly and difficult to scale up, making them unsuitable for industrial applications. Using water as a solvent, the extraction solution will contain more impurities, increasing the difficulty of subsequent purification processes. By comprehensively comparing the extraction efficiency, safety, and ease of industrial application, an organic solvent extraction method was selected using an ethanol aqueous solution as the solvent to extract glycitin [13]. At present, instrumental chemical methods/techniques of analysis used in the detection, identification, and quantification of isoflavones and thus glycitin, including liquid chromatography, gas chromatography, UV absorption method, and ultra high (UHPLC). UHPLC can be used for the determination of soybean isoflavones due to its operation, high sensitivity, high recovery rate, and rapid detection.

## 4. Structure and Physical Properties of Glycitin

**Figure 2:**
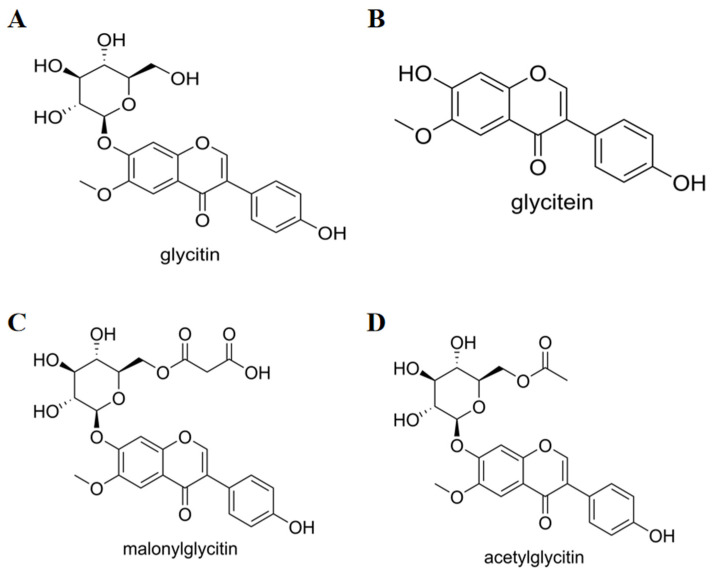
Structural diagram. (**A**) glycitin (**B**) glycitein (**C**) malonylglycitin (**D**) acetylglycitin.

Glycitin is a type of polyphenolic hydroxylated compound. Its core structure consists of rings A, B, and C (Figure 2), with the B ring attached to the third carbon of the C ring serving as the center of antioxidant activity [21]. There is a methoxy group present on the sixth carbon, hence it is also referred to as 6-methoxyglycitin [22]. From a structure–function relationship perspective, its structure is similar to estrogen in mammals and humans. They can bind to estrogen receptors alpha and beta in the body, exerting estrogen-like and anti-estrogenic effects.

Glycitin, usually colorless crystals, is a kind of large isoflavone that has optical activity. The presence of hydroxyl groups in the structure of glycitin affects its solubility [23]. Generally, glycitin is insoluble in water, chloroform, and other solvents, but it is soluble in weak polar solvents such as dimethyl sulfoxide and acetone. Glycitin exhibits stable properties under normal storage conditions, and it has strong thermal stability at temperatures below 150 °C [24].

## 5. Pharmacokinetics in the Body

**Figure 3:**
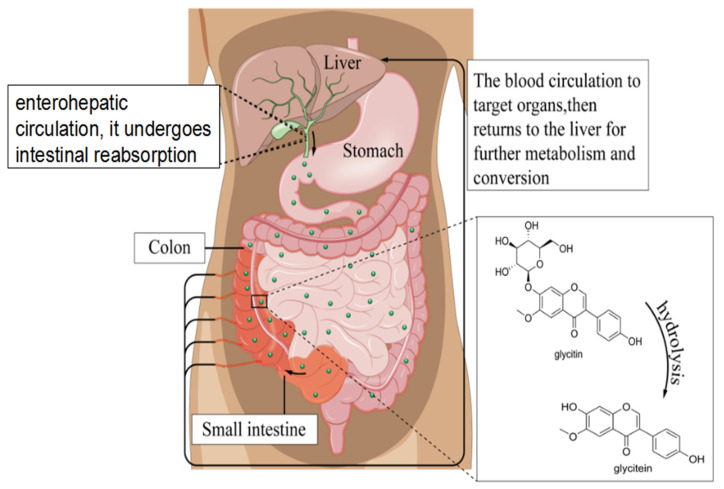
Hydrolysis reaction of glycitin.

Free glycitin can be directly absorbed and exerts its effects in the small intestine, but glycitin needs to be converted into free glycosides under the action of β-glucosidase produced by gut macrobiotic for absorption [25]. Research has shown that the maximum peak of active glycitin can be detected in plasma around 7–8 h after ingestion, and the concentration of glycitin detected in plasma is consistent with the hydrolysis rate of bound glycosides [26]. Glycitin undergoes hydrolysis in the body, primarily through the hydrolysis of the β-glycosidic bond by colonic bacteria, resulting in the removal of the glycosyl and the conversion to free glycitin (Figure 3), which can then be absorbed by the gastrointestinal mucosa. A small portion of free glycitin can also be directly absorbed by the human body. The metabolism of glycitin in the body is complex and follows three main pathways: first, it is transported through the blood circulation to target organs, then returns to the liver for further metabolism and conversion; second, it undergoes enterohepatic circulation; and third, it undergoes intestinal reabsorption [27,28,29]. Glycitin that is not absorbed in the intestine is excreted through feces, while absorbed glycitin is excreted through urine [30]. Research has shown that free glycitin is mostly absorbed in the intestine, with an absorption efficiency of 20–50%, and may have a direct effect on intestinal cells [31]. In addition, studies have found that only about 1–2% of free glycitin enters the circulatory system in humans, while most glycitin is metabolized by intestinal epithelial cells, indicating that free glycitin may exert its maximum function in the intestine [32]. Research has shown that the interaction between free glycitin and gut microbiota can produce estradiol, which is a highly estrogenic binary phenol with a structure highly similar to endogenous estrogen molecules [33,34]. It can replace estrogen and bind to ER-β receptors on the surface of target organ cells without the toxic side effects of exogenous estrogen. In addition, mitochondrial dysfunction occurs in gastrointestinal diseases, including inflammatory bowel diseases and colorectal cancer. An amount of 100–400 mg/kg of soy isoflavones can increase the gene expression of mitochondrial ETC complex II- and IV-related genes SDH (succinate dehydrogenase) and COX, recovering mitochondrial function [35]. Daidzein can promote mitochondrial biosynthesis and the expression of ETC-related genes Tfam, COX1, and cytochrome b (Cytb) in the C_2_C_12_ muscle cell line [36]. Genistein can restore neuronal mitochondrial function, reduce mitochondrial ROS levels, and prevent the release of Cytc from mitochondria into the cytoplasm in a mouse model of cerebral ischemia [37]. However, few studies have focused on the role of glycitin in gut health.

## 6. Biological Activities of Glycitin

### 6.1. Estrogenic and Anti-Estrogenic Activities

**Figure 4:**
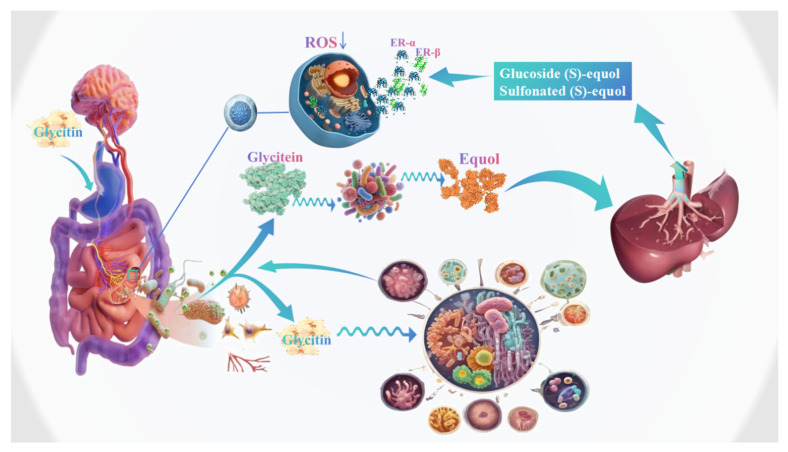
Protection of the human intestinal system from glycitin.

Estrogen, as an endogenous active substance essential for maintaining female physical and mental health, requires the presence of corresponding alpha-estrogen receptors (ER-α) and beta-estrogen receptors (ER-β) to mediate its effects [38]. ER-α is mainly distributed in tissues such as the breast and uterus, while ER-β is commonly found in the central nervous system, bones, ovaries, adrenal glands, and urinary system. Computer simulation methods results showed that soy isoflavones had the ability to bind to the endoplasmic reticulum and can activate estrogen receptors (ER) as plant estrogens [39]. Further studies have shown that under the same conditions, the estrogen-like effect of glycitein is stronger than that of genistin and daidzein [40]. When the estrogen level in the human body is low, the phenolic ring structure of glycitin, which is similar to that of estradiol, can bind to ER and exert weak estrogenic effects. This mechanism can prevent and treat related symptoms that occur in women after menopause due to a decrease in estrogen levels. However, the anti-estrogenic effect was stimulated at high concentrations or too much endogenous estrogen. When the estrogen level in the human body is high, glycitin competitively occupies estrogen receptors and also exhibits weak estrogenic activity, but its activity is only 2% of endogenous estrogen, thus reducing the level of endogenous estrogen [41,42] (Figure 4).

This bidirectional regulatory effect of glycitin not only has significant preventive effects on diseases caused by excessive hormone secretion but also effectively improves estrogen deficiency when it binds to ER-β in target tissues such as the nervous system. In summary, the natural estrogenic and anti-estrogenic effects of glycitin have important implications for the treatment of many chronic diseases, especially in breast cancer and osteoporosis.

### 6.2. Protection of the Human Intestinal System

The influence of gut microbiota on glycitin active components can be mainly divided into the following two types: (1) Gut microbiota promotes the direct digestion and absorption of glycitin active components. It not only provides nutrients for gut microbiota, promotes gut microbiota colonization and proliferation, but also maintains the stability of gut microbiota. (2) The active components of glycitin are catalyzed by glycoside hydrolases secreted by gut microbiota, such as Bifidobacterium and Lactobacillus, to convert glycitin into equol, and enter the hepatic and intestinal circulation (Figure 4) [43]. Research has shown that gut microbiota secrete enzymes, and these enzymes are the key to the biotransformation of glycitin. The number of genes encoded by gut microbiota in healthy adults is more than 150 times that of the human body’s own genes [44]. The diverse gene resources and enzyme systems encoded by gut microbiota endow gut microbiota with multifunctionality in metabolism, such as Bacteroidetes and Firmicutes bacteria in the gut, such as Enterococcus, Lactobacillus, Bifidobacterium, and Clostridium, contain abundant genes encoding glycoside hydrolases, and these can metabolize various glycoside components. β-glucosidase is the most extensively studied hydrolytic enzyme. After entering the colon, glycitin was hydrolyzed by *β-glucosidase Clostridium* sp. *TM-40*, *Escherichia coli HGH2*, *Eubacterium ramulus Julong 601*, *Eggerthella* sp. *YY7918*, *Slackia isoflavoniconvertens DSM 22006* [45,46]. It can be seen that the glycosides in soybean active components that cannot be directly digested and absorbed by the gastrointestinal tract can be biotransformation into new substances with significantly increased biological activity under the action of gut microbiota, thereby promoting human health. However, not all individuals who consume glycitin produce equol. Only approximately one-third to one-half of the population is able to metabolize glycitin to equol. This high variability in equol production is presumably attributable to interindividual differences in the composition of the intestinal microflora, which may play an important role in the mechanisms of action of glycitin [47]. Research has shown that strains related to equol metabolism have been isolated from feces of humans, mice, pigs, and monkeys, including *Adlercreutzia equolifaciens*, *Asaccharobacter celatus*, *Enterorhabdus mucosicola*, *Slackia isoflavoniconvertens*, and *Slackia equolifaciens* [47,48,49]. Therefore, future studies are aimed at identifying the specific bacterial species and strains that are capable of converting glycitin to equol or increasing equol production.

### 6.3. Antioxidant Activity

**Figure 5:**
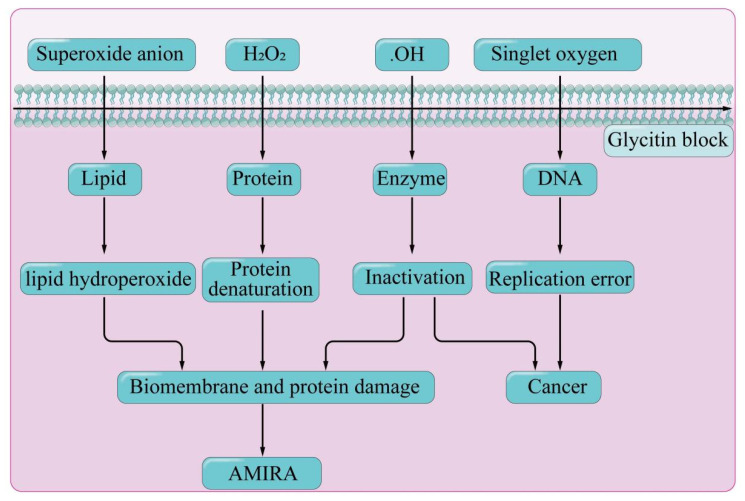
Antioxidant effect of glycitin.

Research in free radical biology has found that many diseases are directly or indirectly related to the toxic reactions of excessive free radicals in the body. Excessive production of free radicals in cells accelerates cellular aging and shortens cell lifespan. Research has shown that black soybean ethanol extract containing glycitin had strong antioxidant activity [50]. In vitro antioxidant experiments confirmed that glycitin, relying on the phenolic hydroxyl groups in its structure, bonded to free radicals and became a stable phenolic radical, thereby clearing reactive oxygen species by inhibiting the oxidation chain [51,52]. In addition, when fruit flies were fed with medium containing 0.5 mg/g of glycitin, the activities of antioxidant enzymes, superoxide dismutase, and glutathione peroxidase (SOD and GSH-Px) in the body significantly increased. Further cell experiments revealed that glycitin cleared intracellular reactive oxygen species and 1,1-diphenyl-2-picrylhydrazyl (DPPH) radicals, inhibited the downstream transcriptional activation of c-Jun N-terminal kinase (JNK), enhanced the activity of antioxidant enzymes, and prevented lipid peroxidation and DNA damage, thereby reducing cell apoptosis [51]. In summary, the antioxidant activity of glycitin was mainly achieved through termination of chain reactions (Figure 5) and enhancement of the activity of antioxidant enzymes. Some studies suggested that the antioxidant capacity of glycitin was weaker compared to antioxidants such as catechin and α-tocopherol [53].

### 6.4. Skin Anti-Aging Activity

The body has a set of anti-aging mechanisms that inhibit the production of free radicals, such as SOD, GSH-PX, Catalase (CAT), glutathione, β-carotene, vitamin C, vitamin E, selenium, zinc, copper, and other trace elements. However, the ability of this mechanism to remove free radicals gradually decreases during the aging process, while the antioxidant activity of glycitin might help the body maintain this clearance ability. A study revealed that a mixture of glycitin and 3-(2,4,6-trimethoxyphenyl)-2,3-dihydro-1H-benzo[f]chromen-1-one (TDB) promoted the proliferation of human skin fibroblasts and reduced wrinkles and melanin production [54]. In addition, cell experiments conducted showed that glycitin induced cell proliferation by stimulating the secretion of transforming growth factor-β (TGF-β) and increased the total collagen, demonstrating significant anti-aging effects [55]. Research has shown that glycitein inhibited matrix metalloproteinase-1 (MMP-1) and increased collagen by downregulating extracellular regulated JNK, protein kinases (ERK), and p38 mitogen-activated protein kinase (MAPK) [56]. Overall, glycitin was considered a potential drug for amelioration skin aging, but the specific anti-aging mechanisms still needed to be studied through in vivo experiments.

### 6.5. Anti-Osteoporosis, Prevention of Osteonecrosis Activity

**Figure 6:**
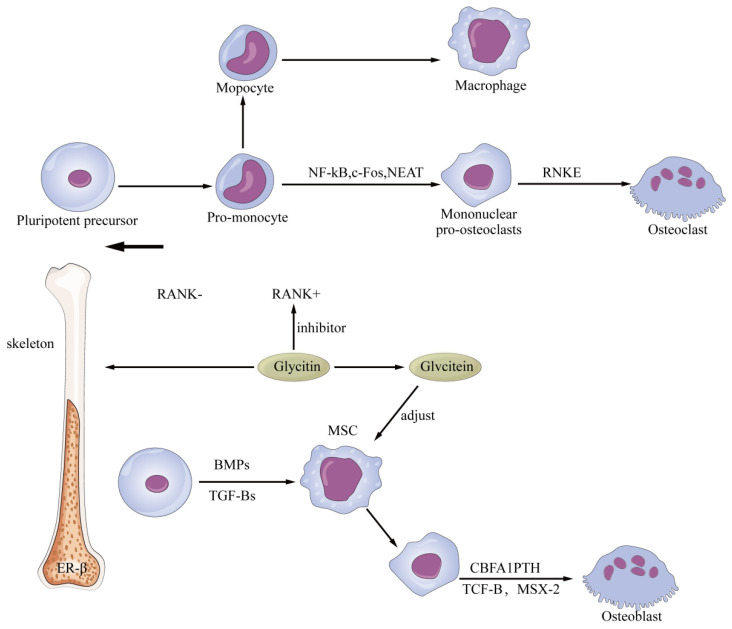
Anti-osteoporosis effect of glycitin.

Osteoporosis (OP) is a systemic chronic bone disease characterized by reduced bone mass, damaged bone structure, and increased bone fragility [57]. Currently, hormone replacement therapy (HRT) with estrogen has become a theoretically effective treatment for osteoporosis, but long-term use of estrogen increases the risk of cancer in women [58]. Due to the lack of safe and effective conventional treatments, many researchers have turned their attention to phytoestrogens such as isoflavones. Research has shown that isolated glycitin from fermented black soybean and found that it had strong anti-osteoporosis activity [59]. Osteoblast differentiation was associated with the expression of bone receptors, and a lack of estrogen during this process increased bone resorption and accelerated bone loss. Research has shown that glycitin was an efficient selective estrogen receptor modulator (SERM) that exhibited agonistic or antagonistic effects when bound to receptors on the bone, while also increasing estrogen levels in the body [60]. In addition, blocking the ER-β receptor in a mouse fracture model experiment not only slowed down osteoporosis but also promoted bone repair [61,62,63]. Therefore, the estrogen-like structure of glycitin competed with estradiol for binding sites on receptors. Glycitin also promoted bone mesenchymal stem cell (BMSC) proliferation and cell formation by inhibiting the expression of TGF-β and protein kinase B [64]. An experiment in vitro had shown that glycitein not only effectively inhibited osteoclast production [65] but also inhibited the differentiation of marrow stromal cells (MSCs) into adipocytes mediated by peroxisome proliferator-activated receptor γ (PPARγ), increasing the number of osteoblasts and the concentration of serum osteocalcin, which was helpful in preventing steroid-induced osteonecrosis [66]. In conclusion, the mechanisms of glycitin in anti-osteoporosis and prevention of osteonecrosis were mainly related to its selective regulation of estrogen receptors and inhibition of TGF-β (Figure 6).

### 6.6. Hypoglycemic and Hypolipidemic Activity

Numerous population studies and animal experiments have shown that soy isoflavones are effective regulators of glucose and lipid metabolism in the body. They played an important role in reducing obesity, lowering the incidence of diabetes, maintaining glucose homeostasis, and increasing insulin sensitivity. Research has shown that soy isoflavone glycosides effectively inhibited lipid absorption and adipocyte differentiation [67]. Soy milk fermented by *Lactobacillus plantarum* HFY01 contained abundant glycitin, which had good weight loss and hypolipidemic effects on mice induced by a high-fat diet [68]. Research demonstrated that glycitin and glycitein had a more significant cholesterol-lowering effect in the body compared to other soy isoflavone components [69]. Research has shown that a hyperlipemia mouse model and found that glycitin reduced lipid oxidation, total cholesterol accumulation, prevented excessive oxidation of low-density lipoprotein, and regulated the release and activity of enzymes related to glucose metabolism, indicating that the antioxidant activity of glycitin was related to reducing lipid accumulation and blood glucose [70]. After the intake of carbohydrates, the human body will absorb and break them down into glucose in the small intestine through α-glucosidase, leading to an increase in postprandial blood sugar levels. Research demonstrated that glycitin obtained from licorice had strong inhibitory activity against α-glucosidase, thereby delaying and reducing the absorption of glucose in the intestinal tract and lowering postprandial blood sugar levels [71]. Glycitin not only reduced enzyme activity to slow down the breakdown of polysaccharides in the body, ameliorated postprandial blood sugar peaks, but also exerted estrogen-like effects to regulate insulin levels and lowered the level of fasting blood sugar. There were already combinations of plant sterols, soy protein, and isoflavones (such as glycitin, biochanin, and puerarin) that effectively lowered total cholesterol and low-density lipoprotein in the body [72]. However, further research is needed on the effects of glycitin on hypoglycemic and hypolipidemic pathways.

### 6.7. Antitumor Activity

**Figure 7:**
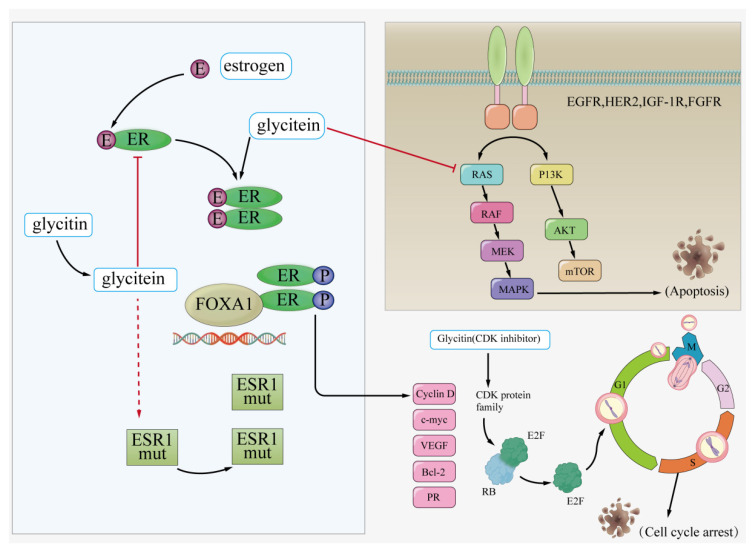
Antitumor effects of glycitin.

One of the main causes of cellular carcinogenesis is the mutation of proto-oncogenes and tumor suppressor genes after DNA damage caused by free radicals [73]. Research has shown that soy isoflavones inhibited tumors in human breast tissue [74]. The phenolic ring structure of glycitin was an effective antimutagen and antioxidant, and it had been proven to have inhibitory effects on cancer development [74]. In a hydrogen peroxide mouse model, after the intake of glycitin, they were converted by intestinal microbial flora into metabolites, including glycitein, which could counteract oxidative stress, eliminate intracellular reactive oxygen species, and inhibit the DNA-binding activity of downstream transcription factor activating protein-1 (AP-1), thus preventing DNA damage [51]. Glycitein induced apoptosis and G0/G1 cell cycle arrest in AGS cells by regulating the MAPK/STAT3/NF-κB signaling pathway related to reactive oxygen species. Research has shown that glycitein has the potential to become a novel targeted therapeutic agent for gastric carcinoma [75]. In addition, research demonstrated that found that glycitein inhibited angiogenesis by reducing the expression of vascular endothelial growth factor (VEGF) and matrix metalloproteinase (MMP), thus inhibiting the formation of vascular tumors [76,77,78]. Further in vivo studies had shown that glycitein affected glioblastoma cells by reducing the activity of matrix metalloproteinase-3 (MMP-3), nuclear transcription factor (NF-κB), and AP-1 [79]. Research has shown that glycitin is an effective inhibitor of lactate dehydrogenase and found that it blocked the conversion of pyruvic acid to lactate dehydrogenase (LDH) in invasive tumor cell glycolysis [80,81]. Glycitin had a certain effect in protecting female breast tissue from excessive estrogen stimulation. Glycitin and glycitein inhibited the adhesion and motility of highly invasive breast carcinoma cells through different signaling pathways [82]. Investigating the effects of glycitein on human breast carcinoma cells, it was found that low concentrations of glycitein damaged cell membranes by increasing membrane permeability, while at high concentrations, it exhibited cytotoxic effects [83]. Using proportional hazards models, it was found that increasing the intake of glycitin and glycitein can reduce the recurrence rate of breast carcinoma by about 60% [84]. However, there was still controversy regarding whether glycitin can be used as a future therapeutic drug for breast carcinoma patients. Some studies suggested that the antioxidant effects of glycitin might help prevent breast carcinoma recurrence, but the estrogen-like effects might increase the risk of breast carcinoma [85]. Further research is needed to determine whether glycitin is harmful to breast carcinoma patients and to elucidate its molecular mechanisms in breast carcinoma development (Figure 7).

### 6.8. Protection of the Human Nervous System

In 2003, researchers found that daily administration of soy isoflavones to menopausal women can significantly improve their oral memory [86]. A report also showed that soy isoflavones have an improving effect on the visual and auditory cognitive function of newly relocated populations in high-altitude areas in 2009 [87]. In 2015, research showed that 65 Alzheimer’s disease (AD) patients were given 100 mg of soy isoflavones daily for 6 months, and language dexterity was enhanced in AD patients [88]. Research has shown that soy isoflavones may enhance the spatial memory ability of rats by upregulating the levels of estradiol, y-aminobutyric acid, and glutamate through soy isoflavones (50, 100, 200 mg/kg) administered orally to rats for 12 months in vivo [34]. Research has shown that the protective effects of soy isoflavones (10, 20, 40 mg/g) administered orally to mice with cognitive impairment induced by Scopolamine (Scop) [89,90]. Both object position recognition and water maze experiments showed a significant improvement in the cognitive ability of the model mice, and this effect was related to promoting the function of the hippocampal cholinergic system and inhibiting hippocampal oxidative stress levels. In addition, Western blot experiments showed that soy isoflavones could significantly upregulate the expression of extracellular signal-regulated kinase (ERK), cAMP response element binding protein (CREB), and BDNF in the hippocampus.

Neurological disorders such as stroke, spinal cord injury, and Parkinson’s disease can occur as a result of damage to the nervous system. Ideal therapeutic drugs for treating these types of diseases should have dual effects of promoting neuronal survival and enhancing regeneration. It was known that glycitin, a transcriptional inducer of arginase 1 (Arginase1, Arg1), possesses these dual effects [91]. Some researchers believed that acetyl glycitin had a significant effect on upregulating choline acetyltransferase (ChAT) and nerve growth factor (NGF) [92]. Studies have shown that glycitin inhibited brain edema and cellular swelling caused by mitochondrial dysfunction through its antioxidant activity, thus protecting brain neurons [93]. Glycitin inhibited cell apoptosis while enhancing the activity of antioxidant enzymes such as GSH, SOD, and CAT, thereby preventing oxidative stress and cell apoptosis induced by rotenone and exerting neuroprotective effects [94]. Glycitin (3 mg/kg and 6 mg/kg; p.o.) treatment for 5 days prevented the depressive effect of reserpine. It also improved the spatial memory at both dose levels. Moreover, in biochemical analysis, glycitein also reduced the brain TBARS and serum tumor necrosis factor-alpha (TNF-α) levels and the potential to manage depression and impaired memory by inhibiting lipid peroxidation and inflammatory stress [95]. These findings suggested that glycitin had promising prospects in the treatment of neurodegenerative diseases such as Parkinson’s disease. In recent years, the efficacy of functional factors such as soy isoflavones in protecting the nervous system and improving cognition has been widely reported. However, recent studies have shown that the biological function of glycitin is mainly attributed to a small molecule compound S-estrol produced by their metabolism in the body. Research has shown that S-estradiol has higher biological activity than glycitin. A survey of Japanese women showed that continuous supplementation with S-equol for 12 weeks significantly improved depression and anxiety symptoms in perimenopausal women. The levels of equol in AD patients were significantly lower than those in the healthy population, indirectly demonstrating that equol may have neuroprotective functions [96]. In addition, Erin et al. found that supplementing high-fat diet mice with equol significantly reduced their exploration time and immobility time in the elevated cross maze and tail suspension experiments, but their mechanism of action was not explored [97]. Johnson et al. used the SH-SY5Y cell model induced by lipopolysaccharide (LPS) to explore the neural mechanism of equol and found that equol can exert a protective effect against oxidative stress damage [98]. S-equol exerts antidepressant and cognitive function improvement effects by alleviating neuroinflammation, combating oxidative stress, inhibiting HPA axis hyperfunction, balancing tryptophan metabolism homeostasis, and regulating monoamine neurotransmitter levels. The above research suggests that equol does have potential neuroprotective functions.

## 7. Conclusions

Since the discovery of glycitin by scientists in 1940, researchers have clarified the active functions of soy isoflavones on human health. Both domestic and international studies had delved into the components of soy isoflavones, and there was ample research supporting the activities of genistin and glycitin. However, research on the biological activities of glycitin, especially acetyl glycitin and propionyl glycitin, is still limited. Currently, epidemiological and animal studies have confirmed that glycitin could prevent and treat human diseases, including carcinoma, neurological disorders, osteoporosis, and menopausal symptoms. However, further research is needed to validate its therapeutic efficacy. Overall, as the extraction and purification processes mature, there will be more breakthrough discoveries regarding the physiological activities of glycitin, and its application fields will continue to expand. The research on the metabolism of glycitin in the body and potential drug interactions lagged behind its pharmacological research, and more clinical studies will be needed in the future to evaluate the potential benefits and risks of glycitin, deepening our understanding of it while discovering new drugs.

## Figures and Tables

**Table 1:** The extraction and purification methods of glycitin derived from soybean.

Pre-Extraction Methods	Extraction Methods	Extraction Conditions	Purification Methods	Reference
-	Extracted with hot water	110 °C and 641 psig (4520 kPa) over 2.3 h of extraction	Solid-phase Amberlite XAD16-HP resin adsorption	[14]
Sonication- assisted extraction	Extracted with hot water	Methanol/water (9:1, *v*/*v*) using sonication for 60 min at room temperature	Acquity UPLC BEH C_18_ column and methanol containing 0.25% (*v*/*v*) acetic acid	[15]
-	Extracted with organic solvent	Methanol/water (6:1, *v*/*v*) using sonication	D_101_ resin-packed column was selected at the bed volume (BV) of 200 mL, feed volume of 3.75 BV, flow rate of 1.5 BV/h	[16]
Microwave- assisted extraction	Extracted with microwave assisted	ACN/water (4:1, *v*/*v*), microwave power of 600 W, extraction time of 1 min	C_18_ high-speed column under isocratic conditions	[17]
-	Extracted with ultrasonic assisted	Solvent: 10 mL 80% EtOH Temperature: 22 °C 80% MeOH, 80% MeOH and 80% EtOH	-	[18]
Ultrasound- assisted extraction	Extracted with ultrasonic assisted	50% ethanol at 60 degrees C using ultrasound-assisted extraction in 20 min.	-	[19]
Pressurize- assisted extraction	Extracted with pressurized solution	methanol (MeOH)/water (80:20, *v*/*v*) in a 75 °C oven for 2 h, and hydrolysis with pressurized solution at room temperature for 10 min	Integration of solid–liquid and liquid–liquid extractions by using aqueous micellar two-phase systems	[20]

## Data Availability

No new data were created or analyzed in this study.

## References

[1] Nakai S., Fujita M., Kamei Y. (2020). Health Promotion Effects of Soy Isoflavones. J. Nutr. Sci. Vitaminol..

[2] Kulprachakarn K., Chaipoot S., Phongphisutthinant R., Paradee N., Prommaban A., Ounjaijean S., Rerkasem K., Parklak W., Prakit K., Saengsitthisak B. (2020). Antioxidant Potential and Cytotoxic Effect of Isoflavones Extract from Thai Fermented Soybean (Thua-Nao). Molecules.

[3] Wu X.J., Zhao L.C., Ma Y.H., Liang W.O., Fang X., Liao Z.L., Zhong Q., Wang J., Wang L. (2022). Analysis of the biotransformation mechanism of soy isoflavones via equol-producing HMA mice model. J. Funct. Foods.

[4] Sekikawa A., Wharton W., Butts B., Veliky C.V., Garfein J., Li J., Goon S., Fort A., Li M., Hughes T.M. (2022). Potential Protective Mechanisms of S-equol, a Metabolite of Soy Isoflavone by the Gut Microbiome, on Cognitive Decline and Dementia. Int. J. Mol. Sci..

[5] Zhou Y., Su Z., Liu G., Hu S., Chang J. (2025). The Potential Mechanism of Soy Isoflavones in Treating Osteoporosis: Focusing on Bone Metabolism and Oxidative Stress. Phytother. Res..

[6] Ohishi T., Miyoshi N., Mori M., Sagara M., Yamori Y. (2022). Health Effects of Soy Isoflavones and Green Tea Catechins on Cancer and Cardiovascular Diseases Based on Urinary Biomarker Levels. Molecules.

[7] Zhou X., Sun H., Tan F., Yi R., Zhou C., Deng Y., Mu J., Zhao X. (2021). Anti-aging effect of Lactobacillus plantarum HFY09-fermented soymilk on D-galactose-induced oxidative aging in mice through modulation of the Nrf2 signaling pathway. J. Funct. Foods.

[8] Van der Eecken H., Joniau S., Berghen C., Rans K., De Meerleer G. (2023). The Use of Soy Isoflavones in the Treatment of Prostate Cancer: A Focus on the Cellular Effects. Nutrients.

[9] Santos Filho L.E.D., Santos G.P.L.D., Silva J.A., Silva F.A., Silva M.N., Almeida A.A., Coqueiro R.D.S., Coimbra C.C., Soares T.J., Magalhães A.C.M. (2022). Dietary Soy Isoflavones Prevent Metabolic Disturbs Associated with a Deleterious Combination of Obesity and Menopause. J. Med. Food.

[10] Lu X., Huang L., Chen J., Ou Y., Wu J., Bodjrenou D.M., Hu J., Zhang Y., Farag M.A., Guo Z. (2024). Marine glycoproteins: A mine of their structures, functions and potential applications. Crit. Rev. Food Sci. Nutr..

[11] Đurović S., Nikolić B., Pisinov B., Mijin D., Knežević-Jugović Z. (2024). Microwave Irradiation as a Powerful Tool for Isolating Isoflavones from Soybean Flour. Molecules.

[12] Valls J., Millán S., Martí M.P., Borràs E., Arola L. (2009). Advanced separation methods of food anthocyanins, isoflavones and flavanols. J. Chromatogr..

[13] Zhang Y., Liu C., Pan Y., Qi Y., Li Y., Li S. (2015). Ultrasound-assisted dynamic extraction coupled with parallel countercurrent chromatography for simultaneous extraction, purification, and isolation of phytochemicals: Application to isoflavones from red clover. Anal. Bioanal. Chem..

[14] An J.H., Ko M.J., Chung M.S. (2023). Thermal conversion kinetics and solubility of soy isoflavones in subcritical water extraction. Food Chem..

[15] Dhaubhadel S., Farhangkhoee M., Chapman R. (2008). Identification and characterization of isoflavonoid specific glycosyltransferase and malonyltransferase from soybean seeds. J. Exp. Bot..

[16] Li H., Liu Y., Jin H., Liu S., Fang S., Wang C., Xia C. (2015). Separation of vitexin-4″-O-glucoside and vitexin-2″-O-rhamnoside from hawthorn leaves extracts using macroporous resins. J. Chromatogr. B.

[17] Terigar B.G., Balasubramanian S., Boldor D., Xu Z., Lima M., Sabliov C.M. (2010). Continuous microwave-assisted isoflavone extraction system: Design and performance evaluation. Bioresour. Technol..

[18] Kim D.H., Yang W.T., Cho K.M., Lee J.H. (2020). Comparative analysis of isoflavone aglycones using microwave-assisted acid hydrolysis from soybean organs at different growth times and screening for their digestive enzyme inhibition and antioxidant properties. Food Chem..

[19] Rostagno M.A., Palma M., Barroso C.G. (2023). Ultrasound-assisted extraction of soy isoflavones. J. Chromatogr..

[20] Cordisco E., Haidar C.N., Coscueta E.R., Nerli B.B., Malpiedi L.P. (2016). Integrated extraction and purification of soy isoflavones by using aqueous micellar systems. Food Chem..

[21] Jung Y.S., Rha C.S., Baik M.Y., Baek N.I., Kim D.O. (2020). A brief history and spectroscopic analysis of soy isoflavones. Food Sci. Biotechnol..

[22] Pereira G.M., Jun S., Li Q.X., Wall M.M., Ho K.K.H.Y. (2023). Formation and physical characterization of soy protein-isoflavone dispersions and emulsions. LWT.

[23] Liu G., Zhou J., Wu S., Fang S., Bilal M., Xie C., Wang P., Yin Y., Yang R. (2024). Novel strategy to raise the content of aglycone isoflavones in soymilk and gel: Effect of germination on the physicochemical properties. Food Res. Int..

[24] Zaheer K., Humayoun Akhtar M. (2017). An updated review of dietary isoflavones: Nutrition, processing, bioavailability and impacts on human health. Crit. Rev. Food Sci. Nutr..

[25] Ma F., Li Z., Liu H., Chen S., Zheng S., Zhu J., Shi H., Ye H., Qiu Z., Gao L. (2024). Dietary-timing-induced gut microbiota diurnal oscillations modulate inflammatory rhythms in rheumatoid arthritis. Cell Metab..

[26] Shinkaruk S., Durand M., Lamothe V., Carpaye A., Martinet A., Chantre P., Vergne S., Nogues X., Moore N., Bennetau-Pelissero C. (2012). Bioavailability of glycitein relatively to other soy isoflavones in healthy young Caucasian men. Food Chem..

[27] Zhao W., Li Y., Cheng X., Wei H., Li P., Fan L., Liu K., Zhang S., Wang H. (2023). The antioxidant Glycitin protects against intervertebral disc degeneration through antagonizing inflammation and oxidative stress in nucleus pulposus cells. Aging.

[28] Islam M.A., Punt A., Spenkelink B., Murk A.J., Rolaf van Leeuwen F.X., Rietjens I.M. (2014). Conversion of major soy isoflavone glucosides and aglycones in in vitro intestinal models. Mol. Nutr. Food Res..

[29] Rüfer C.E., Kulling S.E. (2016). Antioxidant Activity of Isoflavones and Their Major Metabolites Using Different in Vitro Assays. J. Agric. Food Chem..

[30] Cai Y.-Z., Sun M., Xing J., Luo Q., Corke H. (2006). Structure-radical scavenging activity relationships of phenolic compounds from traditional Chinese medicinal plants. Life Sci..

[31] Setchell K.D., Brown N.M., Zimmer-Nechemias L., Brashear W.T., Wolfe B.E., Kirschner A.S., Heubi J.E. (2022). Evidence for lack of absorption of soy isoflavone glycosides in humans, supporting the crucial role of intestinal metabolism for bioavailability. Am. J. Clin. Nutr..

[32] Masilamani M., Wei J., Sampson H.A. (2012). Regulation of the immune response by soybean isoflavones. Immunol. Res..

[33] Sandini T.M., Reis-Silva T.M., Moreira N., Bernardi M.M., Lebrun I., Spinosa H.S. (2019). Effects of isoflavones on behavior, estradiol, glutamate, and GABA levels in intact middle-aged female rats. Nutr. Neurosci..

[34] Zhang W., Jia J., Yang Y., Ye D., Li Y., Li D., Wang J. (2025). Estradiol metabolism by gut microbiota in women’s depression pathogenesis: Inspiration from nature. Front. Psychiatry.

[35] Rasbach K.A., Schnellmann R.G. (2018). Isoflavones promote mitochondrial biogenesis. J. Pharmacol. Exp. Ther..

[36] Yoshino M., Naka A., Sakamoto Y., Shibasaki A., Toh M., Tsukamoto S., Kondo K., Iida K. (2015). Dietary isoflavone daidzein promotes Tfam expression that increases mitochondrial biogenesis in C2C12 muscle cells. J. Nutr. Biochem..

[37] Lee Y.M., Choi J.S., Kim M.H., Jung M.H., Lee Y.S., Song J. (2006). Effects of dietary genistein on hepatic lipid metabolism and mitochondrial function in mice fed high-fat diets. Nutrition.

[38] Pinkerton J.V., Conner E.A. (2019). Beyond estrogen: Advances in tissue selective estrogen complexes and selective estrogen receptor modulators. Climacteric.

[39] Mudrikatin S. (2018). The in silico study of phytoestrogenic activity of soy in substitution of estrogen function. J. Complement. Med. Res..

[40] Gray S.L., Lackey B.R. (2018). Optimizing a recombinant estrogen receptor binding assay for analysis of herbal extracts. J. Herbal. Med..

[41] Srivastava D.P., Woolfrey K.M., Liu F., Brandon N.J., Penzes P. (2010). Estrogen Receptor β Activity Modulates Synaptic Signaling and Structure. J. Neurosci..

[42] Gui Z., Shi W., Zhou F., Yan Y., Li Y., Xu Y. (2025). The role of estrogen receptors in intracellular estrogen signaling pathways, an overview. J. Steroid Biochem. Mol. Biol..

[43] Lee D.H., Kim M.J., Ahn J., Lee S.H., Lee H., Kim J.H., Park S.H., Jang Y.J., Ha T.Y., Jung C.H. (2017). Nutrikinetics of Isoflavone Metabolites After Fermented Soybean Product (Cheonggukjang) Ingestion in Ovariectomized Mice. Mol. Nutr. Food Res..

[44] Mayo B., Vázquez L., Flórez A.B. (2019). Equol: A Bacterial Metabolite from The Daidzein Isoflavone and Its Presumed Beneficial Health Effects. Nutrients.

[45] Xu J., Chen H.B., Li S.L. (2017). Understanding the Molecular Mechanisms of the Interplay Between Herbal Medicines and Gut Microbiota. Med. Res. Rev..

[46] Kawada Y., Yokoyama S., Yanase E., Niwa T., Suzuki T. (2016). The production of S-equol from daidzein is associated with a cluster of three genes in Eggerthella sp. YY7918. Biosci. Microbiota Food Health.

[47] Li M., Han X., Sun L., Liu X., Zhang W., Hao J. (2024). Indole-3-acetic acid alleviates DSS-induced colitis by promoting the production of R-equol from Bifidobacterium pseudolongum. Gut Microbes.

[48] Schröder C., Matthies A., Engst W., Blaut M., Braune A. (2023). Identification and expression of genes involved in the conversion of daidzein and genistein by the equol-forming bacterium Slackia isoflavoniconvertens. Appl. Environ. Microbiol..

[49] Gaya P., Peirotén Á., Medina M., Landete J.M. (2016). Isoflavone metabolism by a collection of lactic acid bacteria and bifidobacteria with biotechnological interest. Int. J. Food Sci. Nutr..

[50] Zheng C., Su Y., Huang X. (2015). Isolation and identification of antioxidant components in ethanol extract of black beans. Food Sci..

[51] Kang K.A., Zhang R., Piao M.J., Lee K.H., Kim B.J., Kim S.Y., Kim H.S., Kim D.H., You H.J., Hyun J.W. (2007). Inhibitory effects of glycitein on hydrogen peroxide induced cell damage by scavenging reactive oxygen species and inhibiting c-Jun N-terminal kinase. Free Radic. Res..

[52] Arifin H.A., Hashiguchi T., Nagahama K., Hashiguchi M., Muguerza M., Sakakibara Y., Tanaka H., Akashi R. (2021). Varietal differences in flavonoid and antioxidant activity in Japanese soybean accessions. Biosci. Biotechnol. Biochem..

[53] Wang F., Yang X., Zhang H., Zhu C., Li L., Wang F. (2018). In vitro Antioxidant Capacities of Isoflavones and Polyphenols in Three Bean Sprouts and Their Effects on Activities of SOD and GSH-Px in Drosophila melanogaster. J. Chin. Inst. Food Sci. Technol..

[54] Ho M.T., Koh D., Cho M. (2014). The mixture of glycitin and TDB (3-(2,4,6-trimethoxyphenyl)-2,3-dihydro-1H-benzo[f]chromen-1-one) could ameliorate skin ageing by anti-wrinkle and anti-melanogenesis effects in dermal fibroblasts and melanocytes. J. Korean Soc. Appl. Biol. Chem..

[55] Kim Y.M., Huh J.S., Lim Y., Cho M. (2015). Soy Isoflavone Glycitin (4′-Hydroxy-6-Methoxyisoflavone-7-D-Glucoside) Promotes Human Dermal Fibroblast Cell Proliferation and Migration via TGF-β Signaling. Phytother. Res..

[56] Seo G.Y., Park S., Huh J.-S., Cho M. (2014). The protective effect of glycitin on UV-induced skin photoaging in human primary dermal fibroblast. J. Korean Soc. Appl. Biol. Chem..

[57] Bjørklund G., Shanaida M., Lysiuk R., Antonyak H., Klishch I., Shanaida V., Peana M. (2022). Selenium: An Antioxidant with a Critical Role in Anti-Aging. Molecule.

[58] Morris G., Talaulikar V. (2023). Hormone replacement therapy in women with history of thrombosis or a thrombophilia. Post. Reprod. Health.

[59] Zhang J., Feng T., Zhang M. (2017). Determination of Isoflavone Content in Light Soy Bean and Study on Its Inhibition of Acetylcholinesterase Activity. Nat. Product. Res. Dev..

[60] Lin C.-C., Chen T.-S., Lin Y.-M., Yeh Y.-L., Li Y.-H., Kuo W.-W., Tsai F.-J., Tsai T.-H., Yen S.-K., Huang C.-Y. (2013). The p38 and NFκB signaling protein activation involved in glycitein protective effects on isoproterenol-treated H9c2 cardiomyoblast cells. J. Funct. Foods.

[61] Liu Y., Wang X., Chang H., Gao X., Dong C., Li Z., Hao J., Wang J., Fan Q. (2018). Mongolian Medicine echinops prevented postmenopausal osteoporosis and induced ER/AKT/ERK pathway in BMSCs. Biosci. Trends.

[62] Zhu H., Jiang J., Wang Q., Zong J., Zhang L., Ma T., Xu Y., Zhang L. (2018). Associations between ERα/β gene polymorphisms and osteoporosis susceptibility and bone mineral density in postmenopausal women: A systematic review and meta-analysis. BMC Endocr. Disord..

[63] Kim M.H., Choi Y.Y., Han J.M., Lee H.S., Hong S.B., Lee S.G., Yang W.M. (2024). Ameliorative effects of Schizandra chinensis on osteoporosis via activation of estrogen receptor (ER)-α/-β. Food Funct..

[64] Winzer M., Rauner M., Pietschmann P. (2010). Glycitein decreases the generation of murine osteoclasts and increases apoptosis. Wien. Med. Wochenschr..

[65] Zhang L., Chen J., Chai W., Ni M., Sun X., Tian D. (2016). Glycitin regulates osteoblasts through TGF-β or AKT signaling pathways in bone marrow stem cells. Exp. Ther. Med..

[66] Li X., Zhang J., Sui S., Yang M. (2005). Effects of genistein, daidzein and glycitein on the osteogenic and adipogenic differentiation of bone marrow stromal cells and on the adipogenic trans-differentiation of osteoblasts. Progress. Nat. Sci. Mater. Int..

[67] Wang S., Wang Y., Pan M.H., Ho C.T. (2017). Anti-obesity molecular mechanism of soy isoflavones: Weaving the way to new therapeutic routes. Food Funct..

[68] Li C., Liu H., Yang J., Mu J., Wang R., Zhao X. (2020). Effect of soybean milk fermented withLactobacillus plantarumHFY01 isolated from yak yogurt on weight loss and lipid reduction in mice with obesity induced by a high-fat diet. RSC Adv..

[69] Lee S.-O., Renouf M., Ye Z., Murphy P.A., Hendrich S. (2007). Isoflavone Glycitein Diminished Plasma Cholesterol in Female Golden Syrian Hamsters. J. Agric. Food Chem..

[70] Zang Y., Igarashi K., Yu C. (2015). Anti-obese and anti-diabetic effects of a mixture of daidzin and glycitin on C57BL/6J mice fed with a high-fat diet. Biosci. Biotechnol. Biochem..

[71] Wu J.Y., Wang T.Y., Ding H.Y., Lee C.C., Chang T.S. (2022). A Novel Soy Isoflavone Derivative, 3′-Hydroxyglycitin, with Potent Antioxidant and Anti-α-Glucosidase Activity. Plants.

[72] Simental-Mendía L.E., Gotto A.M., Atkin S.L., Banach M., Pirro M., Sahebkar A. (2018). Effect of soy isoflavone supplementation on plasma lipoprotein(a) concentrations: A meta-analysis. J. Clin. Lipidol..

[73] Marc-Olivier T., Nicholas J.S.P., George P. (2018). DNA Damage, Repair, and Cancer Metabolism. Front. Oncol..

[74] Kubatka P., Mojžiš J., Pilátová M., Péč M., Kruzliak P., Ullah M.F., Ahmad A. (2016). Soy Isoflavones in the Breast Cancer Risk: From Preclinical Findings to Clinical Strategy. Critical Dietary Factors in Cancer Chemoprevention.

[75] Zang Y., Feng Y., Luo Y., Zhai Y., Ju X., Feng Y., Wang J., Yu C., Jin C. (2019). Glycitein induces reactive oxygen species-dependent apoptosis and G0/G1 cell cycle arrest through the MAPK/STAT3/NF-κB pathway in human gastric cancer cells. Drug Dev. Res..

[76] Patel D.K. (2023). Therapeutic Potential of a Bioactive Flavonoids Glycitin from Glycine max: A Review on Medicinal Importance, Pharmacological Activities and Analytical Aspects. Curr. Tradit. Med..

[77] Cayetano-Salazar L., Olea-Flores M., Zuñiga-Eulogio M.D., Weinstein-Oppenheimer C., Fernández-Tilapa G., Mendoza-Catalán M.A., Zacapala-Gómez A.E., Ortiz-Ortiz J., Ortuño-Pineda C., Navarro-Tito N. (2021). Natural isoflavonoids in invasive cancer therapy: From bench to bedside. Phytother. Res. PTR.

[78] Kaufman-Szymczyk A., Jalmuzna J., Lubecka-Gajewska K. (2025). Soy-derived isoflavones as chemo-preventive agents targeting multiple signalling pathways for cancer prevention and therapy. Br. J. Pharmacol..

[79] Lee E.J., Kim S.Y., Hyun J.W., Min S.W., Kim D.H., Kim H.S. (2010). Glycitein inhibits glioma cell invasion through down-regulation of MMP-3 and MMP-9 gene expression. Chem.-Biol. Interact..

[80] Li S., Li S., Liu C., Liu C., Zhang Y. (2017). Extraction and isolation of potential anti-stroke compounds from black soybean (*Glycine max*, L. Merrill) guided by in vitro, PC12 cell model. J. Funct. Foods.

[81] Rani R., Kumar V. (2015). Recent Update on Human Lactate Dehydrogenase Enzyme 5 (hLDH5) Inhibitors: A Promising Approach for Cancer Chemotherapy. J. Med. Chem..

[82] Valachovicova T., Slivova V., Bergman H., Shuherk J., Sliva D. (2004). Soy isoflavones suppress invasiveness of breast cancer cells by the inhibition of NF-kappaB/AP-1-dependent and -independent pathways. Int. J. Oncol..

[83] Zhang B., Su J., Bai Y., Li J., Liu Y. (2015). Inhibitory effects of O-methylated isoflavone glycitein on human breast cancer SKBR-3 cells. Int. J. Clin. Exp. Pathol..

[84] Boutas I., Kontogeorgi A., Dimitrakakis C., Kalantaridou S.N. (2022). Soy Isoflavones and Breast Cancer Risk: A Meta-analysis. Vivo.

[85] Liu C., Zhao J., Liu J., Wang Y. (2025). Innovating non-small cell lung cancer treatment with novel TM-GL/NPs nanoparticles for Glycitin delivery. Cell Biol. Toxicol..

[86] Smeriglio A., Calderaro A., Denaro M., Laganà G., Bellocco E. (2019). Effects of Isolated Isoflavones Intake on Health. Curr. Med. Chem..

[87] Li R., Robinson M., Ding X., Geetha T., Al-Nakkash L., Broderick T.L., Babu J.R. (2022). Genistein: A focus on several neurodegenerative diseases. J. Food Biochem..

[88] Gleason C.E., Fischer B.L., Dowling N.M., Setchell K.D., Atwood C.S., Carlsson C.M., Asthana S. (2015). Cognitive Effects of Soy Isoflavones in Patients with Alzheimer’s Disease. J. Alzheimer’s Dis..

[89] Lu C., Gao R., Lv J., Chen Y., Li S., Zhang L., Zhang N., Wang Y., Fan B., Liu X. (2020). Neuroprotective effects of soy isoflavones on chronic ethanol-induced dementia in male ICR mice. Food Funct..

[90] Lu C., Wang Y., Wang D., Zhang L., Lv J., Jiang N., Fan B., Liu X., Wang F. (2018). Neuroprotective Effects of Soy Isoflavones on Scopolamine-Induced Amnesia in Mice. Nutrients.

[91] Tang Z.J., Huang Z.P., Yang W.J., Zou Y.X., Cai J.P. (2014). Effect of Daidzein intravitreal injection on optic nerve injury in rats. Int. Eye Sci..

[92] Prajapati R., Park S.E., Park H.J., Jung H.A., Choi J.S. (2021). Identification of a Potent and Selective Human Monoamine Oxidase-A Inhibitor, Glycitein, an Isoflavone Isolated from Pueraria lobata Flowers. ACS Food Sci. Technol..

[93] Zielonka J., Gebicki J., Grynkiewicz G. (2003). Radical scavenging properties of genistein. Free Radic. Biol. Med..

[94] Dong N., Yang Z.L. (2022). Glycitein exerts neuroprotective effects in Rotenone-triggered oxidative stress and apoptotic cell death in the cellular model of Parkinson’s disease. Acta Biochim. Pol..

[95] Diksha, Singh L. (2024). Glycitein prevents reserpine-induced depression and associated comorbidities in mice: Modulation of lipid peroxidation and TNF-α levels. Naunyn-Schmiedeberg’s Arch. Pharmacol..

[96] Viña J., Borrás C., Mas-Bargues C. (2024). Genistein, A Phytoestrogen, Delays the Transition to Dementia in Prodromal Alzheimer’s Disease Patients. J. Alzheimer’s Dis..

[97] Bax E.N., Cochran K.E., Mao J., Wiedmeyer C.E., Rosenfeld C.S. (2019). Opposing effects of S-equol supplementation on metabolic and behavioral parameters in mice fed a high-fat diet. Nutr. Res..

[98] Xicoy H., Wieringa B., Martens G.J. (2017). The SH-SY5Y cell line in Parkinson’s disease research: A systematic review. Mol. Neurodegener..

